# Productivity loss due to polycystic ovary syndrome and its relationship to race, mental health and healthcare delivery indices

**DOI:** 10.1016/j.xfre.2024.02.004

**Published:** 2024-02-15

**Authors:** Heather Gibson Huddleston, Alexander Milani, Rachel Blank

**Affiliations:** a Department of Obstetrics, Gynecology and Reproductive Sciences, University of California-San Francisco, San Francisco, California; b Allara, New York, New York

**Keywords:** PCOS, productivity loss, race, mental health, healthcare delivery

## Abstract

**Objective:**

To study the impact of polycystic ovary syndrome (PCOS) on work-related impairments and explore relationships with race, mental health, and healthcare delivery indices.

**Design:**

A cross-sectional internet-based survey.

**Setting:**

North American women with PCOS between August 2022 and October 2022.

**Patients:**

Individuals with a self-reported diagnosis of PCOS.

**Intervention(s):**

Not applicable.

**Main Outcome Measures:**

The primary outcome was missed work because of PCOS. The secondary outcomes included leave from work, impacts on the quality of work, and feelings of being held back at work because of PCOS.

**Results:**

Of 1,105 respondents, 1,058 reported having PCOS diagnosed by a physician. Of this group, 50.4% reported missing work because of PCOS, 72% felt that PCOS impacted the quality of their work, and 51.5% felt held back at work by PCOS. Multivariate analyses revealed that missing work because of PCOS was independently associated with black race, lack of insurance, requiring multiple doctors for a PCOS diagnosis, needing ≥3 doctors for current care, decreased satisfaction with care, and symptoms of anxiety and depression.

**Conclusions:**

Polycystic ovary syndrome significantly impacts employment-related productivity. Factors such as race, mental health, and healthcare delivery appear to play a crucial role in the extent of this impact.

Polycystic ovary syndrome (PCOS) is a common and multifaceted syndrome that impacts approximately 10% of women (1). PCOS is diagnosed on the basis of the presence of oligomenorrhea, hyperandrogenism, and/or polycystic ovary morphology; however, the syndrome presents additional health challenges for affected women, including infertility, metabolic dysregulation and diabetes, depression, and anxiety. The metabolic and hormonal disturbances, as well as the associated symptoms, are chronic, persisting to varying degrees throughout the reproductive years (2, 3, 4, 5), with adverse impacts on health-related quality of life (6).

For women in young adulthood—a critical period for establishing a professional career and achieving financial stability—work experience and productivity may be an important measure of general well-being. A variety of chronic gynecologic health disorders have been linked to challenges in work experience and productivity, including primary dysmenorrhea, endometriosis, and fibroids (7, 8, 9). Concerningly, it is likely that health-related impacts to work experiences, particularly during the early career years, could have long-term effects on career trajectory.

The degree to which PCOS symptoms may interfere with work performance has not previously been described, representing a significant gap in the literature. Therefore, we aimed to describe employment-related outcomes in a cohort of individuals with PCOS, including self-reported measures of absenteeism and work quality. Additionally, we aimed to uncover potential contributors to work disruption in PCOS. Recent reports have highlighted broad dissatisfaction with PCOS medical care among patients with PCOS (10), and thus, we hypothesized that patients reporting dissatisfaction in their evaluation and management would be more likely to report adverse work outcomes.

## Materials and methods

### Ethical Considerations

This study was conducted in adherence to ethical guidelines and received full approval from the Brany Institutional Review Board on August 22, 2022 (institutional review board number, IRB00000080). The principles of voluntary participation and anonymity were observed throughout the study. Participants were not required to provide any personal identifiers, thus ensuring complete anonymity. The completion of the survey was understood as an informed consent from the participants, implying their willingness to partake in the study and share their experiences.

### Study Design, Setting, and Participants

This study was designed as a cross-sectional survey administered to a community-based sample of women from the United States and Canada, identified and recruited via advertisements disseminated through social media and virtual care waitlist emails. Advertisements requested participants with PCOS for an online research study estimated to take 20 minutes. No remuneration was provided; however, participants were entered to win a $250.00 gift certificate. The inclusion in analyses described herein required a self-reported previous diagnosis of PCOS by a healthcare provider.

### Instruments

For work outcomes, we used dichotomous questions adapted from a previously published survey regarding work and endometriosis (11):

• Have you missed work because of symptoms related to your PCOS?
• Have you ever taken an extended sick leave because of your PCOS?
• Have symptoms of PCOS affected the quality of your work?
• Have symptoms related to your PCOS held you back from achieving your career goals?

To gauge the satisfaction with the diagnostic experience and care for PCOS, we used a set of questions adapted from surveys employed in a previous international study (10). Symptoms of depression and/or anxiety were assessed using a modified Patient Health Questionnaire, modified to exclude 1 question regarding suicidality (12), and the Generalized Anxiety Disorder 7-item scale (13).

### Statistics

The primary outcome of interest was reporting missed work; the secondary outcomes of interest were reporting extended leave, having quality of work affected, and feeling held back at work. The initial univariate logistic regression analyses were performed to obtain crude odds ratios (ORs) and 95% confidence intervals (CIs). These analyses helped identify relationships between our primary outcomes of interest (missing work, extended leave, quality of work affected, and feeling held back in career) and potential predictor variables. Multivariate logistic regression models were then constructed, incorporating all predictors that had a significant association with the primary outcome (missing work) at a significance level of *P* < .05. Statistical analyses were performed using Stata (StataCorp, College Station, TX). The amount of missing data for predictor variables was minimal (<5% for all of variables). Missing data for predictor variables were assumed to be random and did not contribute to summary data. For multivariate analyses, observations were dropped when missing data were present.

## Results

The first objective of this study was to ascertain the impact of PCOS on various aspects of employment. A total of 1,105 respondents participated in the study, 1,058 of whom confirmed a previous PCOS diagnosis by a healthcare professional and constituted the final cohort for analysis. The clinical and demographic characteristics are shown in Table 1. The mean age of the participants was 30.6 (standard deviation, ± 6.2; range, 15–57) years, and they represented a broad range of racial/ethnic groups, with the majority being white (57.5%). The educational levels varied across the cohort—25.5%, 37.4%, and 14.8% reported having advanced, college, and associate degrees, respectively, whereas 22.3% reported having high school education or less.

**Table 1:** Clinical and demographic characteristics of study population.

Characteristic	Mean ± SD or % (n)
**Clinicaland demographic characteristics (n)**	
Age (1,038), y	30.6 ± 6.2
Race (1,058), %	
*White*	57.5% (612)
*Black*	7.1% (76)
*Asian*	12.1% (128)
*Hispanic*	19.1% (203)
*Other*	3.7% (39)
Educational level (1,057), %	
*High school or less*	22.3% (236)
*Associate degree*	14.8% (156)
*College graduate*	37.4% (395)
*Advanced degree*	25.5% (270)
Income (1,053), %	
*<40,000*	21.3% (244)
*40,000–79,999*	31.9% (336)
*80,000–120,000*	21.8% (229)
*>120,000*	25.1% (264)
Employment status (1,057), %	
*Full time*	70.0% (740)
*Part time*	7.6% (80)
*Unemployed*	9.3% (99)
*Student*	4.5% (47)
*Student and working*	8.5% (90)
Insurance status (1,053), %	
*Commercial HMO*	27.4% (288)
*Commercial PPO*	51.3% (540)
*Government*	13.2% (139)
*Military*	1.1% (12)
*None*	7.0% (74)
BMI, kg/m^2^ (1,050)	34.8 ± 8.9
Moderate-severe anxiety symptoms (1,058), %	50.7% (536)
Moderate-severe depression symptoms (1,058), %	48.4% (512)
Age of symptom onset (1,034), y	18.0 ± 5.4
Age of diagnosis (1,010), y	23.0 ± 6.0
**PCOS care experience**	
Professionals seen before diagnosis (1,054), %	
*1*	27.0% (285)
*2*	25.9% (273)
*3 or more*	47.1% (496)
No. of doctors seen in past 3 years for the management of symptoms, %	
*0*	0.76% (8)
*1*	26.2% (277)
*2*	26.9% (284)
*≥3*	46.2% (488)
Told by a provider who you wanted to see that it was not covered (n = 1,056)	
*Yes*	28.3% (299)
*No*	71.7% (757)
Satisfaction with PCOS diagnostic experience, % (n = 1,057)	
*Very satisfied*	7.9% (83)
*Somewhat satisfied*	19.0% (201)
*Neutral*	20.6% (218)
*Somewhat dissatisfied*	27.1% (286)
*Very dissatisfied*	25.5% (269)
Satisfaction with PCOS care (1055), %	
*Very satisfied*	4.9% (52)
*Somewhat satisfied*	12.3% (130)
*Neutral*	15.3% (161)
*Somewhat dissatisfied*	31.2% (329)
*Very dissatisfied*	36.3% (383)
**Employment outcomes related to PCOS (n = 1,058)**	
Missed work (1058), %	
*No*	49.6% (525)
*Yes*	50.4% (533)
Extended leave (1056), %	
*No*	83.2% (879)
*Yes*	16.7% (177)
Quality of work impacted (1058), %	
*No*	28.0% (298)
*Yes*	72.0% (767)
Feeling held back at work (1053), %	
*No*	48.5% (511)
*Yes*	51.5% (542)

*Note:* Data are shown as means (standard deviations) unless otherwise indicated. BMI = body mass index; HMO = health maintenance organization; PCOS = polycystic ovary syndrome; PPO = preferred provider organization.

In terms of employment status, a majority were employed full time (70%). We found that over half of the respondents experienced work-related disruptions because of their condition. Specifically, 50.4% of the women reported missing work because of PCOS-related symptoms, and 16.7% required an extended leave because of their condition. The perceived quality of work was also negatively impacted, with 72% of participants reporting this outcome and 51.5% reporting that they felt held back at work by PCOS symptoms. The univariate associations between work outcome predictors of interest are detailed in Table 2.

**Table 2:** Univariate associations with employment outcomes.

Characteristic	Missed work (OR [CI])	Extended leave (OR [CI])	Quality impacted (OR [CI])	Held back (OR [CI])
Age (y)	1.01 (0.99–1.03)	1.00 (0.98–1.03)	0.96 (0.94–0.98)^c^	0.97 (0.95–0.99)^b^
Race				
White	Ref	Ref	Ref	Ref
Asian	0.64 (0.40–1.05)	0.97 (0.49–1.90)	0.90 (0.54–1.51)	1.01 (0.62–1.62)
Black	1.54 (1.04–2.27)^a^	2.32 (1.49–3.61)^c^	1.12 (0.73–1.71)	1.22 (0.83–1.78)
Hispanic	0.96 (0.70–1.32)	1.03 (0.66–1.60)	1.28 (0.89–1.83)	1.28 (0.93–1.76)
Other	0.76 (0.40–1.47)	1.25 (0.54–2.91)	1.62 (0.76–3.44)	0.86 (0.45–1.65)
Educational level				
High school	Ref	Ref	Ref	Ref
Associate degree	0.83 (0.55–1.25)	0.86 (0.52–1.42)	1.07 (0.68–1.68)	0.95 (0.63–1.44)
College degree	0.53 (0.38–0.73)^c^	0.58 (0.38–0.89)^a^	1.01 (0.70–1.45)	0.76 (0.05–1.05)
Advanced degree	0.65 (0.46–0.93)^a^	0.65 (0.41–1.02)	0.86 (0.58–1.60)	0.54 (0.38–0.77)^c^
Income				
<40,000	Ref	Ref	Ref	Ref
40,000-79,999	0.65 (0.46–0.92)^a^	0.46 (0.29–0.70)^c^	1.01 (0.68–1.49)	0.84 (0.60–1.19)
80,000–120,000	0.63 (0.44–0.92)^a^	0.66 (0.42–1.04)	0.72 (0.48–1.10)	0.54 (0.37–0.79)
>120,000	0.69 (0.48–0.99)^a^	0.40 (0.25–0.65)^c^	0.64 (0.43–0.95)^a^	0.57 (0.39–0.81)^b^
Employment				
Full time	Ref	Ref	Ref	Ref
Part time	1.11 (0.70–1.77)	1.36 (0.77–2.40)	1.09 (0.65–1.84)	0.96 (0.60–1.52)
Unemployed	1.28 (0.84–1.97)	0.40 (0.12–1.31)	1.38 (0.65–2.94)	1.08 (0.58–2.02)
Student	0.66 (0.37–1.20)	1.23 (0.62–2.45)	1.02 (0.56–1.85)	1.16 (0.67–1.99)
Student/employed	1.18 (0.76–1.80)	1.17 (0.55–2.46)	0.71 (0.39–1.31)	0.67 (0.37–1.20)
Insurance				
PPO	Ref	Ref	Ref	Ref
HMO	0.86 (0.64–1.14)	1.08 (0.72–1.61)	0.86 (0.64–1.22)	0.97 (0.73–1.29)
Medicare/Medicaid	1.11 (0.76–1.61)	2.04 (1.30–3.21)^b^	1.60 (0.76–2.55)^a^	1.37 (0.91–2.06)
Military	0.50 (0.15–1.68)	0.55 (0.07–4.29)	0.81 (0.24–2.74)	0.49 (0.15–1.67)
None	2.36 (1.40–4.00)^b^	2.22 (1.26–3.92)^b^	1.36 (0.77–2.42)	1.72 (1.01–2.91)^a^
Age at diagnosis (y)	0.99 (0.97–1.01)	1.00 (0.97–1.03)	1.00 (0.98–1.03)	1.00 (0.99–1.02)
Years for diagnosis (>3 y)	1.28 (1.00–1.64)	1.40 (1.01–1.94)^a^	1.26 (0.96–1.66)	1.09 (1.01–1.18)
Told not covered	1.46 (1.11–1.10)^b^	1.80 (1.28–2.52)^c^	2.15 (1.06–1.82)^a^	0.60 (0.46–0.79)^c^
≥3 providers for diagnosis	2.06 (1.61–2.63)^c^	2.20 (1.58–3.07)^c^	1.63 (1.24–2.14)^c^	1.70 (1.33–2.17)
≥3 providers for care in 3 y	1.14 (1.05–1.24)^b^	1.36 (0.98–1.88)	1.39 (1.07–1.29)^c^	1.21 (0.96–1.55)
Very dissatisfied with diagnostic experience	1.75 (1.32–2.31)^c^	1.92 (1.36–2.70)^c^	2.07 (1.47–2.93)^c^	1.47 (1.11–1.94)
Very dissatisfied with medical care	1.82 (1.41–2.35)^c^	1.62 (1.16–2.24)^b^	1.72 (1.28–2.30)^c^	1.58 (1.22–2.03)^c^
BMI (kg/m^2^)	1.02 (1.00–1.03)^a^	1.00 (0.98–1.02)	1.00 (0.99–1.02)	1.00 (0.99–1.02)^b^
Moderate-severe anxiety symptoms^d^	2.33 (1.82–2.98)^c^	3.33 (2.49–4.45)^c^	2.30 (1.75–3.09)^c^	2.87 (2.23–3.80)^c^
Moderate-severe depression symptoms^e^	2.12 (1.66–2.71)^c^	2.14 (1.53–2.99)^c^	3.33 (2.49–4.45)^c^	3.67 (2.85–4.74)^c^

*Note:* Age (years), age at diagnosis (years), and BMI (kg/m^2^) were treated as continuous variables. BMI = body mass index; CI = confidence interval; HMO = health maintenance organization; OR = odds ratio; PPO = preferred provider organization.

a *P* < .05.

b *P* < .01.

c *P* < .001.

d Defined as a Generalized Anxiety Disorder 7-item scale score of >9.

e Defined as a Patient Health Questionnaire score of >14.

### Multivariate Analyses

#### Missing work

Multivariate analyses used complete data from a total of 1,022 subjects and revealed several independent sociodemographic associations (Table 3): black race slightly increased the odds of missing work because of PCOS symptom (adjusted OR [aOR], 1.67; 95% CI, 1.07–2.59; *P* < .05), whereas having a college education was protective compared with having only completed high school (aOR, 0.56; 95% CI, 0.374–0.822; *P* < .05). Compared with preferred provider organization insurance, government insurance reduced the odds of missing work, whereas having no insurance doubled the odds (aOR, 2.04; 95% CI, 1.08–3.83; *P* < .05). Factors associated with PCOS care were also linked to missing work: respondents who reported that they required ≥3 doctors to arrive at a PCOS diagnosis were twice as likely to miss work (aOR, 2.01; 95% CI, 1.52–2.66; *P* < .001) compared with respondents who required <2 years, and those who reported being very dissatisfied with their PCOS care were 55% more likely to miss work than those who were more satisfied (*P* < .01). The presence of moderate or severe anxiety also doubled the risk of missing work (*P* < .001).

**Table 3:** Multivariate associations with employment outcomes.

Characteristic	Missed work (aOR [CI])	Extend leave (aOR [CI])	Work quality (aOR [CI])	Held back (aOR [CI])
Age (y)	1.01 (0.99–1.03)	1.10 (0.98–1.04)^a^	0.97 (0.94–0.99)^a^	0.98 (0.96–1.00)
Race				
White	Ref	Ref	Ref	Ref
Asian	0.72 (0.42–1.23)	1.15 (0.56–2.38)	0.93 (0.53–1.64)	1.09 (0.64–1.86)
Black	1.67 (1.07–2.60)^a^	2.54 (1.53–4.22)^c^	0.93 (0.53–1.64)	1.19 (0.76–1.85)
Hispanic	0.95 (0.67–1.37)	0.98 (0.60–1.57)	1.12 (0.74–1.68)	1.10 (0.76–1.59)
Other	0.87 (0.67–1.37)	1.49 (0.60–3.60)	1.78 (0.79–4.02)	1.17 (0.57–2.39)
Educational level				
High school	Ref	Ref	Ref	Ref
Associate degree	0.88 (0.56–1.39)	1.03 (0.59–1.79)	1.31 (0.78–2.17)	1.15 (0.73–1.82)
College degree	0.56 (0.37–0.82)^b^	0.74 (0.45–1.22)	1.19 (0.77–1.83)	0.95 (0.64–1.41)
Advanced degree	0.72 (0.47–1.11)	0.81 (0.48–1.41)	1.15 (0.73–1.84)	0.67 (0.44–1.04)
Income				
<40,000	Ref	Ref	Ref	Ref
40,000–79,999	0.66 (0.44–1.00)^a^	0.61 (0.37–1.01)	1.16 (0.73–1.85)	0.90 (0.60–1.37)
80,000–120,000	0.70 (0.44–1.11)	0.96 (0.55–1.67)	0.79 (0.47–1.32)	0.61 (0.38–0.97)
>120,000	0.86 (0.54–1.37)	0.69 (0.38–1.23)	0.90 (0.54–1.50)	0.81 (0.51–1.29)
Told by your provider that it is not covered	1.36 (1.00–1.847)	1.44 (0.99–2.09)	1.67 (1.16–2.40)^b^	1.35 (0.99–1.84)
Insurance				
PPO	Ref	Ref	Ref	Ref
HMO	0.84 (0.61 1.15)	1.10 (0.71–1.68)	0.74 (0.52–1.04)	0.86 (0.62–1.18)
Government	0.56 (0.35–0.90)^a^	1.21 (0.69–2.11)	1.06 (0.61–1.84)	0.78 (0.48–1.26)
Military	0.29 (0.07–1.21)	0.62 (0.07–5.28)	0.84 (0.21–3.44)	0.46 (0.12–1.71)
None	2.04 (1.08–3.83)^a^	1.92 (0.99–3.75)	1.13 (0.57–2.23)	1.57 (0.85–2.89)
≥3 providers seen for care (compared with <3 providers)	1.24 (0.93–1.64)	1.49 (1.03–2.17)^a^	1.38 (1.02 1.88)^a^	1.34 (1.01–1.79)
≥3 providers seen for diagnosis (compared with <3 providers)	2.01 (1.52–2.66)^c^	1.49 (1.03–2.17)^c^	1.54 (1.13–2.10)^b^	1.83 (1.38–2.43)^c^
Very dissatisfied with medical care^a^	1.54 (1.14–2.08)^b^	1.20 (0.82–1.76)	1.23 (0.88–1.72)	1.30 (0.96–1.76)
Very dissatisfied with diagnostic experience^d^	1.17 (0.83–1.65)^c^	1.32 (0.88–2.00)	1.72 (1.14–2.58)^a^	1.07 (0.75–1.52)
Moderate-severe anxiety symptoms^e^	2.01 (1.46–2.77)^c^	2.13 (1.37–3.30)^b^	1.28 (0.90–1.82)	1.60 (1.16–2.20)^b^
Moderate-severe depression symptoms^f^	1.35 (0.98–1.87)	1.30 (0.85–1.99)	2.49 (1.73–3.58)^c^	2.47 (1.79–3.41)^c^
BMI (kg/m^2^)	1.01 (1.00–1.03)	1.00 (0.98–1.02)	1.00 (0.99–1.02)	1.00 (0.99–1.02)

*Note:* Age (years), age at diagnosis (years), and BMI (kg/m^2^) were treated as continuous variable. aOR = adjusted odds ratio; BMI = body mass index; CI = confidence interval; HMO = health maintenance organization; PPO = preferred provider organization.

a *P* < .05.

b *P* < .01.

c *P* < .001.

d Compared with somewhat dissatisfied, neutral, somewhat satisfied, or very satisfied.

e Defined as a Generalized Anxiety Disorder 7-item scale score of >9.

f Defined as a Patient Health Questionnaire score of >14.

#### Leave

Requiring an extended leave was less common, impacting only 16.7% of respondents. Similar factors that were associated with missed work were associated with requiring an extended leave, including black race (aOR, 2.54; 95% CI, 1.5–4.2; *P* < .001), requiring ≥3 providers for care (aOR, 1.49; 95% CI, 1.03–2.17 *P* < .001), and anxiety (aOR, 2.13; 95% CI, 1.4–3.3; *P* < .001). In addition, requiring ≥3 different providers for care increased the odds of an extended leave by 49% (aOR, 1.49; 95% CI, 1.03–2.17; *P* < .05).

#### Quality of work

Reporting that PCOS had negative impacts on quality of work was common, with 72% of respondents reporting this outcome. Increasing age was protective, suggesting that concern over work quality may disproportionately impact those early in their career. Although insurance status was not associated with this outcome, being told by a provider who you wanted to see that it was not covered by your insurance was linked to work quality impacts. Several factors regarding the PCOS care experience also demonstrated relationships with work quality, including requiring ≥3 providers for diagnosis (aOR, 1.54; 95% CI, 1.13–2.10; *P* < .01), requiring ≥3 providers for current care (aOR, 1.38; 95% CI, 1.02–1.88; *P* < .05), and being very dissatisfied with the diagnostic experience (aOR, 1.72; 95% CI, 1.14–2.58; *P* < .05). Finally, depressive symptoms were associated with more than doubled odds of work quality being impacted.

#### Held back at work because of PCOS

Approximately one half of respondents felt that they were held back at work by PCOS symptoms. Factors independently associated with this outcome included requiring ≥3 doctors for diagnosis (aOR, 1.83; 95% CI, 1.38–2.43; *P* < .001) and both moderate-severe anxiety (aOR, 1.60; 95% CI, 1.16–2.20; *P* < .01) and depression symptoms (aOR, 2.47; 95% CI, 1.79–3.41; *P* < .001).

## Discussion

PCOS is a highly prevalent chronic condition, impacting approximately 10% of reproductive-aged females (1). The disorder gives rise to a wide array of symptoms, encompassing gynecologic, dermatologic, metabolic, and psychiatric concerns, translating, ultimately, to reduced health-related quality of life (6). One’s ability to participate in professional life, unencumbered by health problems, is an element of quality of life that has not been specifically explored in PCOS. Using the results of a survey of over 1,000 North American individuals with PCOS, we found that PCOS symptoms adversely impact employment for a significant proportion of those with this common disorder. To our knowledge, this is the first report to describe how PCOS symptoms adversely shape one’s employment experience, either because of absenteeism, impacts on work quality, or simply feeling held back in professional attainment. Because PCOS symptoms manifest beginning in adolescence and are often at their most severe in young adulthood, a critical period for career establishment, our findings raise concerns about the negative and lasting effects of PCOS on professional trajectory and attainment. Viewed at scale, productivity loss by a percentage of individuals with this common disorder adds to the already substantial economic burden of PCOS (14).

We found that several social determinants of health, including race and educational level, were related to adverse professional outcomes. Notably, we found that black women had a higher odds of missing work and requiring extended leave, independent of income or insurance status. Disparities in the lived experience of PCOS across racial groups in the United States is not well described; however, our findings align with a growing body of literature showing that black women have persistently poorer outcomes related to obstetric and gynecologic health (15, 16, 17) and experience higher levels of depression (4) and lower fertility-related quality of life (18). Against this backdrop, our results demonstrating disproportionate impacts of PCOS on employment outcomes for black women may indicate inadequacies in the treatment of PCOS symptoms for this group and highlights a need for additional efforts to clarifying and improve the experience of black women with PCOS.

We found that several measures of healthcare access and satisfaction were robustly related to adverse work-related outcomes. Those without health insurance were more likely to miss work, and even when controlling for insurance status, and respondents who were told that they could not see a provider of choice were more likely to report decreased work quality. Moreover, dissatisfaction with PCOS diagnostic experience and care, as well as the need to consult multiple providers for the same, demonstrated negative associations across all employment outcomes. Broad dissatisfaction with PCOS care has previously been reported (10, 19). In the context of a healthcare environment designed to approach disease within narrowly defined, siloed specialties, the multifaceted and multisystem nature of PCOS likely contributes to patients “falling through the cracks,” with no one specialty perfectly suited to provide comprehensive care. In addition, the heterogeneous presentation and existence of multiple, evolving diagnostic schemes have likely generated provider confusion. Indeed, a recent survey of gynecologists and reproductive endocrinologists reported significant knowledge gaps regarding diagnostic criteria and common comorbidities (20). Our demonstration that inadequate PCOS care has downstream implications for professional attainment further emphasizes the need to improve provider education around evidence-based, internationally accepted consensus guidelines (21, 22). More broadly, the international PCOS guidelines have called for a greater emphasis on integrated and comprehensive PCOS care models, although it is likely that implementation remains a challenge (21, 23).

Finally, and not surprisingly, we noted independent associations between symptoms of depression and anxiety and work outcomes. The high prevalence of mental health disorders among women with PCOS is now well described and is closely tied to quality-of-life indicators (24, 25, 26). Nevertheless, mental health comorbidities in PCOS remain underrecognized by the healthcare providers (20). Our results suggest that increased recognition and management of this domain of the PCOS experience could yield better professional outcomes for affected women.

This study has several important strengths, including the size and diversity of the sample and the novelty of our findings. Although this is the first report of PCOS and work outcomes, our findings are consistent with research showing that other gynecologic conditions, including endometriosis, menstrual disturbance, and fibroids, have the potential to negatively impact women’s work productivity (7, 8, 27). Although our study provides valuable insights, it has certain limitations. The reliance on survey respondents recruited through social media and PCOS virtual care waitlists may select for subjects with more significant PCOS concerns, and thus, the results may not be generalizable to all women with PCOS. Furthermore, the cross-sectional design precludes the assessment of causality, and the reliance on self-report may introduce recall bias. Our employment questions were adapted from previous work but were not validated in a PCOS population. These questions did not allow for quantification of absenteeism, nor did they elicit information regarding the reasons for work disruptions. Future work, including through the use of both quantitative instruments and qualitative studies, may provide deeper insights regarding our findings. Finally, with no control group included, we were unable to determine how these work experiences may compare to those with and without other gynecologic disorders. Studies using community-based cohort of participants with and without PCOS would further expand our understanding of intersections between reproductive health and professional success in the population at large.

## Conclusion

This study describes a significant burden experienced by people with PCOS in the workplace. Although our study design precludes assignment of causality, we did identify important associations that suggest potential targets that may improve the employment experiences of people with PCOS, including expanded access to comprehensive PCOS care and a greater recognition of the mental health burden of this common disorder.

## CRediT Authorship Contribution Statement

**Heather Gibson Huddleston:** Writing – review & editing, Writing – original draft, Formal analysis, Data curation, Conceptualization. **Alexander Milani:** Writing – review & editing, Project administration, Data curation. **Rachel Blank:** Writing – review & editing, Resources, Project administration, Conceptualization.

## Declaration of Interests

H.H. has nothing to disclose. A.M. has nothing to disclose. R.B. has nothing to disclose.
